# Functional Characterization of a Novel OTU-like Deubiquitinase from *Neospora caninum* and Discovery of Small-Molecule Inhibitors

**DOI:** 10.3390/ijms27125178

**Published:** 2026-06-07

**Authors:** Fatih Kocabaş, Sezer Akgöl, Pınar Siyah

**Affiliations:** 1 Department of Molecular Biology and Genetics, Faculty of Engineering and Natural Sciences, Istanbul Atlas University, Istanbul 34403, Türkiye; 2 Medizinische Klinik und Poliklinik I, LMU University Hospital, 80336 Munich, Germany; sezer.akoel@med.uni-muenchen.de; 3 Department of Biochemistry, School of Pharmacy, Bahcesehir University, Istanbul 34353, Türkiye; pinar.siyah@med.bau.edu.tr

**Keywords:** *Neospora caninum*, neosporosis, OTU-like deubiquitinase, ncOTU, small molecule inhibitors, host immune evasion, ubiquitin

## Abstract

*Neospora caninum* is a major apicomplexan pathogen responsible for significant reproductive losses in livestock, yet lacks effective therapeutics. Here, we identify and functionally characterize a previously unstudied OTU-like deubiquitinase (ncOTU; XP_003886403) as a key modulator of host–pathogen interactions. Sequence and structural analyses revealed conservation of the catalytic triad (D257, C260, H362) and a Y305-W315-G316 inhibition pocket analogous to viral OTU proteases. Recombinant ncOTU exhibited robust deubiquitinase activity and significantly reduced global ubiquitination levels in mammalian cells, preferentially targeting mono-ubiquitinated and low-molecular-weight substrates. Transcriptomic analysis demonstrated that ncOTU expression correlates with suppressed NF-κB signaling, type I interferon responses, and downstream antiviral effectors, while partially uncoupling upstream nucleic acid sensing pathways. Structure-based virtual screening and biochemical validation identified multiple small-molecule inhibitors targeting the conserved inhibition pocket. Dose-response analysis revealed submicromolar potency for ncOTUi-9 (IC_50_ = 0.1 μM), ncOTUi-8 (0.2 μM), and ncOTUi-19 (0.3 μM), whereas ncOTUi-3 showed lower activity (7.1 μM). Interaction analyses confirmed stable binding within the inhibition pocket, with more extensive contact networks correlating with increased potency. Collectively, these findings establish ncOTU as a functional deubiquitinase that contributes to evasion and highlight it as a promising therapeutic target for neosporosis.

## 1. Introduction

*Neospora caninum* is an obligate intracellular protozoan parasite belonging to the phylum Apicomplexa. Neosporosis is recognized as a major cause of infectious abortion in cattle worldwide, leading to significant economic losses in the livestock industry [1]. In Türkiye, the parasite is widely endemic, with recent nationwide studies reporting a herd-level seroprevalence of 27.5% in dairy farms based on bulk tank milk samples collected across seven geographical regions [2]. In addition, *N. caninum* causes neuromuscular disease in dogs, the definitive host, and has been reported in other animals including sheep, goats, and horses [3,4,5]. Despite its global distribution and economic impact, no effective vaccines or approved specific therapeutic agents are currently available for the treatment or prevention of neosporosis [6].

Intracellular pathogens, including apicomplexan parasites, have sophisticated mechanisms to subvert host cellular defenses. A critical aspect of successful intracellular parasitism is the ability to antagonize host innate immune signaling pathways, particularly the NF-κB and type-I interferon (IFN) pathways, which are essential for initiating antiviral and antiparasitic responses [7,8]. One elegant strategy employed by certain viruses and intracellular parasites involves the expression of deubiquitinating enzymes (DUBs) that remove ubiquitin (Ub) and Ub-like modifications from host signaling proteins [9,10,11].

Ubiquitination is a reversible post-translational modification that regulates numerous cellular processes, including protein degradation, signal transduction, DNA repair, and immune signaling [12,13]. Deubiquitinating enzymes (DUBs) reverse these modifications and have emerged as critical modulators of host-pathogen interactions [14]. The Ovarian Tumor (OTU) family of DUBs is of particular interest because several viruses, including the Crimean-Congo Hemorrhagic Fever Virus (CCHFV), encode OTU domain-containing proteases that suppress host innate immunity by deconjugating ubiquitin and ISG15 from host proteins [9]. The CCHFV OTU protease has been extensively characterized, and its inhibition pocket (Y89-W99) has been identified as a promising target for antiviral drug development [9,10].

Recent bioinformatics analyses have revealed the presence of putative OTU-like domains in several apicomplexan parasites, including Plasmodium species and *Neospora caninum* [15]. Subsequent studies have confirmed that OTU deubiquitinases are expanded in apicomplexan parasites, with comprehensive characterization of these enzymes recently performed in *Toxoplasma gondii* [16]. While some *N. caninum* OTU-like proteins have been predicted bioinformatically, the functional characterization of these enzymes has remained largely unexplored. Importantly, no studies have investigated the potential OTU-like protein in *N. caninum* (accession number XP_003886403), which we have designated ncOTU.

In this study, we hypothesized that ncOTU functions as a genuine deubiquitinating enzyme that contributes to *N. caninum* pathogenesis by modulating host immune responses. To test this hypothesis, we expressed and purified recombinant ncOTU, characterized its enzymatic activity *in vitro* and *in vivo*, investigated its effects on host cellular ubiquitination and immune gene expression, and identified and validated small molecule inhibitors targeting ncOTU. Our results provide the first functional characterization of ncOTU as a *N. caninum* OTU deubiquitinase and establish it as a promising therapeutic target for neosporosis.

## 2. Results

### 2.1. Identification and Sequence Analysis of ncOTU

**Figure 1:**
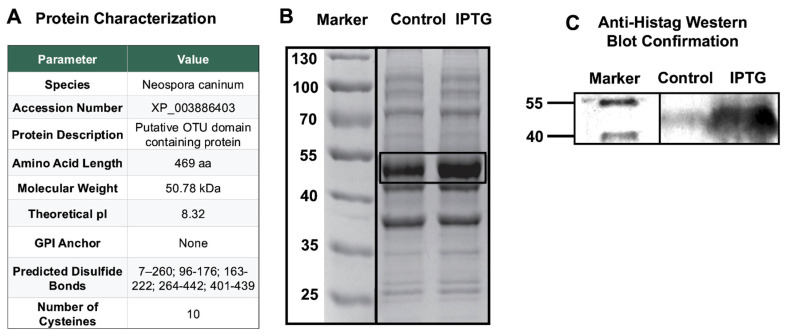
Recombinant expression of ncOTU protein. (**A**) Summary table of the physicochemical properties of *Neospora caninum* OTU-like protein (ncOTU; XP_003886403) (**B**) SDS-PAGE analysis of ncOTU expression in *E. coli* BL21(DE3) cells. Lane 1: Uninduced control; Lane 2: IPTG-induced. The box indicates the expected band at approximately 50.8 kDa. (**C**) Anti-HisTag Western blot confirmation of ncOTU expression. Uninduced control; IPTG-induced. The blot shows specific recognition of the HisTag on recombinant ncOTU [17].

Bioinformatics analysis of the *N. caninum* genome revealed a putative OTU domain-containing protein (XP_003886403), which we designated ncOTU. Sequence analysis showed that ncOTU is a 469-amino acid protein with a predicted molecular weight of 50.78 kDa and a theoretical pI of 8.32 (Figure 1A). No GPI anchor was predicted, and 10 cysteine residues were identified with potential disulfide bond formation. Multiple sequence alignment revealed that ncOTU shares significant homology with the CCHFV OTU protease, particularly within the catalytic and inhibition regions (Table 1). Strikingly, the catalytic triad residues (D257, C260, H362) and the inhibition pocket residues (Y305, W315, G316) were 100% identical at the catalytic triad positions (D257/D37, C260/C40, H362/H151) and the three inhibition pocket residues (Y305/Y89, W315/W99, G316/G100) when aligned.

### 2.2. Homology Modeling of ncOTU

Since no crystal structure is available for ncOTU, we generated a three-dimensional homology model using the CCHFV OTU structure (PDB ID: 3PRP.A) as a template (Figure S1A). The resulting model showed excellent structural alignment with the template, with an RMSD value of 1.459 Å and a sequence alignment score of 0.085. Ramachandran plot analysis indicated that >95% of residues were in favored regions, confirming the structural validity of the model (Figure S1A).

Examination of the ncOTU model revealed a well-defined catalytic pocket containing the conserved catalytic triad (D257, C260, H362) (Figure S1B). Importantly, the Y305-W315-G316 inhibition pocket was positioned adjacent to the catalytic site, forming a surface-accessible cavity suitable for small molecule binding (Figure S1B). Molecular docking of human ubiquitin (PDB ID: 3PHW.B) into the ncOTU model demonstrated that ubiquitin binds within the conserved Y-W pocket, with the C-terminal tail of ubiquitin positioned near the catalytic cysteine (C260) (Figure S1C), consistent with the binding mode observed in CCHFV OTU-ubiquitin complexes.

### 2.3. Cloning, Expression, and Purification of Recombinant ncOTU

The codon-optimized ncOTU gene (1437 bp) was successfully synthesized and cloned into pET-26b(+) using NdeI and NcoI restriction sites. Restriction digestion with NdeI and EcoRI confirmed the presence of the correct insert (Figure S2A,B). DNA sequencing verified the nucleotide sequence with no mutations (Figure S2C,D).

Recombinant ncOTU was expressed in *E. coli* BL21(DE3) cells following IPTG induction at 25 °C. SDS-PAGE analysis revealed a prominent band at approximately 50 kDa in induced lysates (Figure 1B). Western blot analysis using anti-HisTag antibody confirmed the identity of the recombinant protein (Figure 1C).

**Figure 2:**
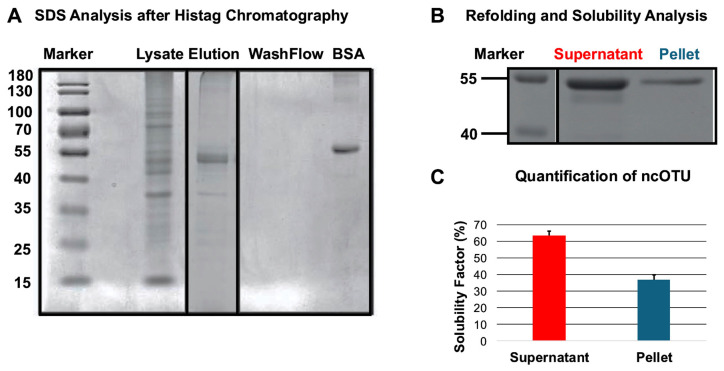
Affinity-based purification and refolding of ncOTU protein. (**A**) SDS-PAGE analysis of fractions collected during HisTrap HP affinity chromatography. Samples include lysate, flow-through, wash, and elution fractions. BSA is shown as a loading control. The purified ncOTU protein is visible in the elution lane at the expected molecular weight. (**B**) Refolding and solubility analysis of purified ncOTU in PBS. The bar graph shows the solubility factor (%) calculated as [supernatant protein]/[supernatant + pellet protein] × 100. Note that ncOTU remained >65% soluble, indicating proper folding. (**C**) SDS-PAGE analysis of soluble (supernatant) and insoluble (pellet) fractions following ncOTU refolding and desalting in PBS, demonstrating the distribution of properly folded versus aggregated protein species [17].

Purification of ncOTU using HisTrap HP affinity chromatography on an ÄKTAprime system yielded a single major elution peak (Figure 2A). SDS-PAGE analysis of eluted fractions showed highly purified ncOTU at >90% homogeneity (Figure 2A). Proper protein folding is essential for enzymatic activity. Solubility analysis in PBS showed that 65–70% of purified ncOTU remained soluble after desalting and refolding (Figure 2B,C). SDS-PAGE analysis confirmed that the soluble fraction contained properly folded ncOTU, while the pellet fraction contained aggregated protein (Figure 2C). This level of solubility was considered sufficient for subsequent functional assays.

### 2.4. ncOTU Exhibits Deubiquitinase Activity In Vitro

**Figure 3:**
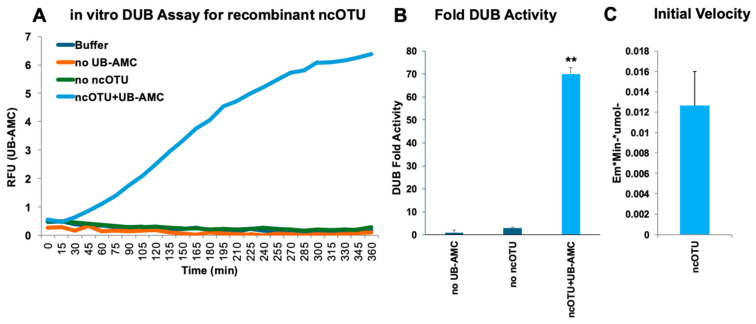
*In Vitro* Deubiquitinating Activity. (**A**) Time-dependent cleavage of UB-AMC by recombinant ncOTU (RFU increase) (**B**) Quantification of fold-activity compared to negative controls (**C**) Initial enzymatic velocity of ncOTU calculated from the linear portion of the progress curve. ** *p* < 0.01 vs. control (no UB-AMC).

To determine whether ncOTU possesses deubiquitinating enzyme activity, we performed fluorometric UB-AMC cleavage assays. Recombinant ncOTU catalyzed time-dependent cleavage of UB-AMC, resulting in increasing fluorescence over 360 min (Figure 3A). Compared to controls, ncOTU exhibited significant DUB activity upto 70 fold (Figure 3B) with an initial enzymatic velocity over 0.012 (Em·Min^−1^·µM^−1^) (Figure 3C). The activity was linear within the first 120 min, establishing optimal conditions for subsequent inhibition studies.

### 2.5. ncOTU Modulates the Cellular Ubiquitome In Vivo

**Figure 4:**
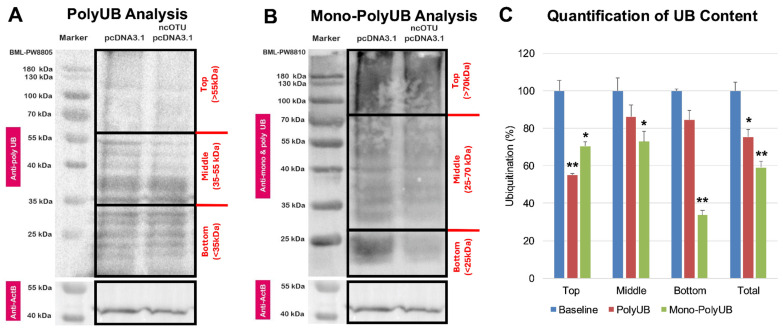
*In vivo* Ubiquitome Analysis. (**A**) Western blot analysis of polyubiquitinated proteins using anti-polyUb antibody (BML-PW8805). HEK293 cells were transfected with empty pcDNA3.1 vector (control) or pcDNA3.1-ncOTU. Beta-actin is shown as a loading control. (**B**) Western blot analysis using anti-mono/polyUb antibody (BML-PW8810) showing the reduction of both mono- and polyubiquitinated proteins in ncOTU-expressing cells. (**C**) Quantification of total ubiquitin signal normalized to beta-actin and control (**Top**, **Middle**, **Bottom**). * *p* < 0.05 or ** *p* < 0.01 vs. control.

To evaluate the deubiquitinase activity of ncOTU in mammalian cells, HEK293 cells were transfected with pcDNA3.1-ncOTU or empty vector control, followed by immunoblot analysis of ubiquitinated proteins (Figure 4A,B). Immunoblotting with an anti-polyubiquitin antibody (BML-PW8805) revealed a global reduction in polyubiquitinated protein levels upon ncOTU expression (Figure 4A). This decrease was most pronounced in the high molecular weight (top; >55 kDa) region, representing heavily polyubiquitinated substrates. Moderate reductions were also observed in the middle (35–55 kDa) and bottom (<35 kDa) fractions. These findings indicate that ncOTU broadly affects polyubiquitin chains, with a stronger impact on higher molecular weight conjugates. Using an antibody that detects both mono- and polyubiquitinated proteins (BML-PW8810), a more extensive reduction in ubiquitination signals was observed (Figure 4B). The decrease was particularly evident in the middle (25–70 kDa) and bottom (<25 kDa) regions, which are enriched in mono-ubiquitinated proteins and short ubiquitin chains. While the top fraction (>70 kDa) also showed reduced signal intensity, the effect was less pronounced compared to lower molecular weight regions, suggesting that ncOTU preferentially targets mono-ubiquitin modifications and shorter ubiquitin chains.

Densitometric analysis confirmed these observations and revealed region-specific differences in ncOTU-mediated deubiquitination (Figure 4C). In the polyubiquitin dataset, ubiquitination levels were reduced by 45% in the top fraction (*p* < 0.01), 10–15% in the middle fraction as well as in in the bottom fraction (not significant), resulting in an overall 25–30% decrease in total polyubiquitin signal (*p* < 0.05). In contrast, the mono/polyubiquitin dataset showed a more pronounced reduction across all regions, with decreases of 30% in the top (*p* < 0.05), 25–30% in the middle (*p* < 0.05), and 60–65% in the bottom fraction (*p* < 0.01). This corresponded to an overall reduction of 40–45% in total ubiquitin signal (*p* < 0.01).

Notably, the most significant decrease was consistently observed in the bottom fraction, indicating that ncOTU preferentially removes mono-ubiquitin or low-molecular-weight ubiquitin conjugates. The middle fraction exhibited moderate reductions with lower statistical significance, whereas the top fraction showed consistent but less pronounced decreases, reflecting partial trimming of high-molecular-weight polyubiquitin chains. Collectively, these data demonstrate that ncOTU functions as an active deubiquitinase *in vivo*, with a preferential impact on mono-ubiquitinated substrates and lower molecular weight ubiquitin species.

### 2.6. ncOTU Correlates with Suppressed Host Innate Immune Gene Expression

**Figure 5:**
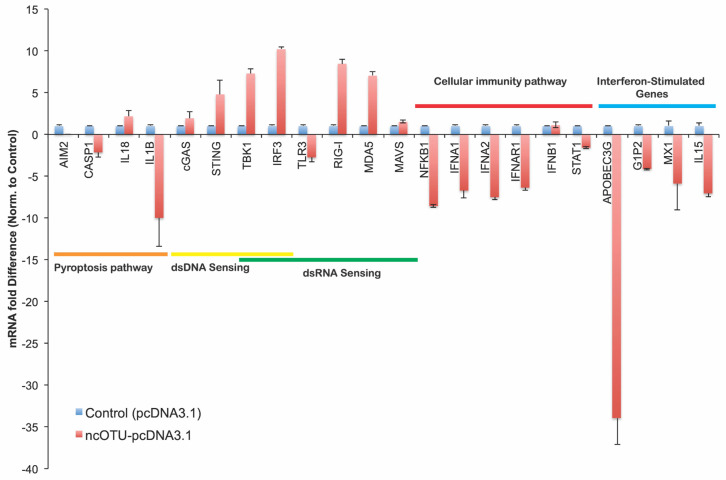
Suppression of Host Immune Gene Expression. Real-time PCR analysis of immune-related genes in HEK293 cells transfected with empty vector (control) or pcDNA3.1-ncOTU. Fold changes are shown relative to control (set to 1). Pyroptosis pathway genes: *AIM2*, *CASP1*, *IL1B*, *IL18*. DNA/RNA sensing pathway genes: *cGAS*, *STING*, *TBK1*, *IRF3*, *TLR3*, *RIG-I*, *MDA5*, *MAVS*. NF-κB and interferon signaling genes: *NFKB1*, *IFNA1*, *IFNA2*, *IFNAR1*, *IFNB1*, *STAT1*. Interferon-stimulated genes: *APOBEC3G*, *G1P2*, *IL15*, *MX1*.

Given that viral OTU proteases antagonize host immunity by suppressing NF-κB and interferon signaling, we examined whether ncOTU similarly modulates these pathways. Real-time PCR analysis of HEK293 cells expressing ncOTU revealed dramatic downregulation of key host innate immune genes (Figure 5).

Notably, ncOTU expression resulted in a differential modulation of innate immune gene networks, revealing a clear separation between upstream sensing and downstream effector responses. Genes associated with inflammasome activation and pyroptosis were generally suppressed. The pyroptosis-associated gene *CASP1* expression decreased by approximately 2-fold, while the pro-inflammatory cytokine *IL1B* showed a pronounced 10-fold reduction. In contrast, *AIM2*, a cytosolic DNA sensor linked to inflammasome activation, remained unchanged. These results suggest that ncOTU does not strongly affect inflammasome sensing capacity but selectively dampens downstream inflammatory execution.

Components of the cytosolic DNA sensing pathway were all upregulated. *cGAS* expression increased by 2-fold, while its adaptor *STING* was elevated by 5-fold. Downstream signaling molecules, including *TBK1* and *IRF3*, were strongly induced, showing 7-fold and 10-fold increases, respectively. This pattern indicates that ncOTU expression is associated with activation of DNA sensing machinery at the transcriptional level. Similarly, genes involved in RNA sensing exhibited activation. *RIG-I* and *MDA5* were upregulated by 9-fold and 7-fold, respectively. In contrast, *TLR3* was reduced by 2-fold, and *MAVS* showed only a modest 1.5-fold increase. These findings suggest partial activation of RNA sensing pathways, with some divergence at the level of adaptor signaling.

Key regulators of downstream immune signaling were markedly suppressed. *NFKB1* expression decreased by 9-fold, indicating inhibition of NF-κB-mediated transcriptional responses. In parallel, type I interferon genes were significantly downregulated, with *IFNA1* reduced by 7-fold, *IFNA2* by 8-fold, and *IFNB1* by 6-fold. Additionally, *IFNAR1*, encoding the interferon receptor, was reduced by 6-fold, further suggesting impaired signal amplification and responsiveness.

A strong suppression of antiviral effector genes was also observed. *APOBEC3G* exhibited the most pronounced decrease, with a 35-fold reduction, while *MX1* and *ISG15* (*G1P2*) were reduced by 9-fold and 7-fold, respectively. The inflammatory cytokine *IL15* was also downregulated by 8-fold. This widespread inhibition of ISGs indicates that ncOTU effectively blocks the execution phase of the interferon response.

Collectively, these findings demonstrate that ncOTU uncouples innate immune sensing from downstream effector activation. While upstream nucleic acid sensing pathways (dsDNA and dsRNA) appear transcriptionally activated, key signaling nodes and antiviral effectors including NF-κB, type I interferons, and ISGs are strongly suppressed. This pattern suggests that ncOTU may preferentially interfere with signal propagation and immune amplification rather than initial nucleic acid sensing in the context of the innate immune pathways examined. Whether ncOTU affects other immune branches (complement, NK cell activity, phagocytosis) remains to be investigated.

### 2.7. Screening Identifies Potential ncOTU Inhibitors

To identify small molecule inhibitors targeting ncOTU, we performed molecular docking of a drug-like compound library against the ncOTU homology model. The docking grid was centered on the conserved Y305-W315-G316 inhibition pocket. A total of 20 compounds were evaluated for their predicted binding affinity toward ncOTU, revealing a broad distribution of docking scores ranging from −5.1 to −7.9 kcal/mol (Table 2). Among these, ncOTUi-12 (−7.9 kcal/mol) and ncOTUi-16 (−7.7 kcal/mol) exhibited the strongest predicted binding, indicating a high likelihood of stable complex formation within the ncOTU catalytic site. These top-performing compounds share structurally complex, polycyclic scaffolds with multiple aromatic rings and heterocyclic moieties, which likely facilitate π–π stacking interactions and enhanced binding stability. Following these, ncOTUi-1 (−7.5 kcal/mol) and ncOTUi-13 (−6.9 kcal/mol) also demonstrated relatively strong binding affinities, suggesting that extended conjugated systems and the presence of functional groups such as amides and halogen substitutions (e.g., bromine in ncOTUi-13) may further stabilize ligand–protein interactions. A second tier of compounds, including ncOTUi-18 (−6.8 kcal/mol), ncOTUi-19 (−6.7 kcal/mol), ncOTUi-7 (−6.7 kcal/mol), and ncOTUi-17 (−6.6 kcal/mol), showed moderate binding affinities, indicating that these scaffolds may serve as promising starting points for further optimization.

Notably, the initially highlighted compounds ncOTUi-11 (−6.4 kcal/mol) and ncOTUi-19 (−6.7 kcal/mol) displayed moderate binding, whereas ncOTUi-16 (−7.7 kcal/mol) clearly ranks among the top candidates. Compounds with simpler or less rigid structures, such as ncOTUi-8 (−5.1 kcal/mol), ncOTUi-3 (−5.8 kcal/mol), and ncOTUi-6 (−5.8 kcal/mol), exhibited comparatively weaker binding, suggesting that reduced aromaticity and structural flexibility may negatively impact binding efficiency.

Overall, the data indicate that binding affinity correlates with structural complexity, aromatic ring density, and the presence of heterocyclic and electron-withdrawing substituents, which likely enhance interactions within the ncOTU active site. Importantly, the observed binding energy distribution and ranking are consistent with those previously reported for malarial OTU [11], supporting the structural and functional conservation between ncOTU and related viral and malarial OTU proteases.

### 2.8. Experimental Validation of ncOTU Inhibitors

**Figure 6:**
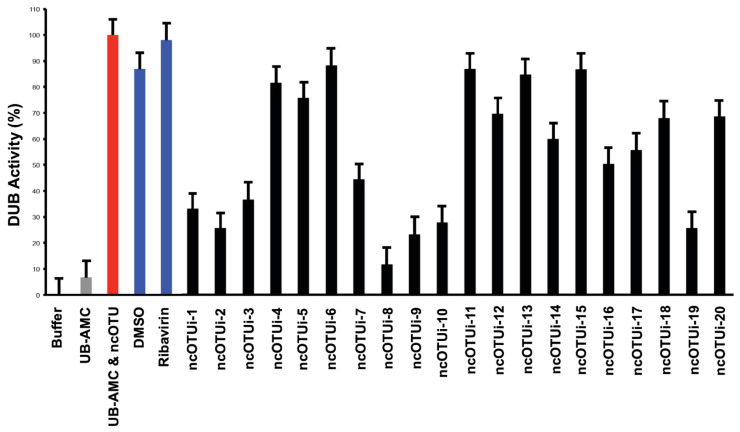
Screening of Small Molecule ncOTU Inhibitors. Screening of small molecule compounds against ncOTU DUB activity at 20 μM concentration. Percent inhibition was calculated relative to untreated control (UB-AMC & ncOTU). DMSO and Ribavirin were used as negative controls.

The 20 compounds were experimentally evaluated for their ability to inhibit ncOTU deubiquitinase activity using the UB-AMC assay at a final concentration of 20 μM (Figure 6). Enzymatic activity was normalized to the ncOTU control (set at 100%), and reductions in activity were interpreted as inhibitory effects. Among the tested compounds, several exhibited substantial inhibition of ncOTU activity. ncOTUi-1 exhibited 67% inhibition, while ncOTUi-2, ncOTUi-3 and ncOTUi-10 reduced ncOTU activity to below 35% (>65% inhibition). ncOTUi-8 was the most potent, with 88% inhibition, followed by ncOTUi-9 (77% inhibition) and ncOTUi-19 (75% inhibition). A second group of compounds demonstrated moderate inhibition. These included ncOTUi-7 (55% inhibition) and ncOTUi-16 (50% inhibition), indicating partial suppression of enzymatic activity. Additional compounds such as ncOTUi-14 (40% inhibition) and ncOTUi-17 (45% inhibition) also showed measurable but less pronounced effects. In contrast, several compounds exhibited weak or minimal inhibitory activity (<35% inhibition at 20 μM), including ncOTUi-4, -5, -6, -11, -12, -13, -15, -18, and -20, consistent to some extent with their lower predicted binding affinities.

Overall, the results demonstrate that a subset of compounds effectively inhibit ncOTU activity *in vitro*, with ncOTUi-8 and ncOTUi-19 emerging as the most potent inhibitors under the tested conditions. Notably, the experimental data only partially correlate with docking predictions, highlighting the importance of empirical validation.

**Figure 7:**
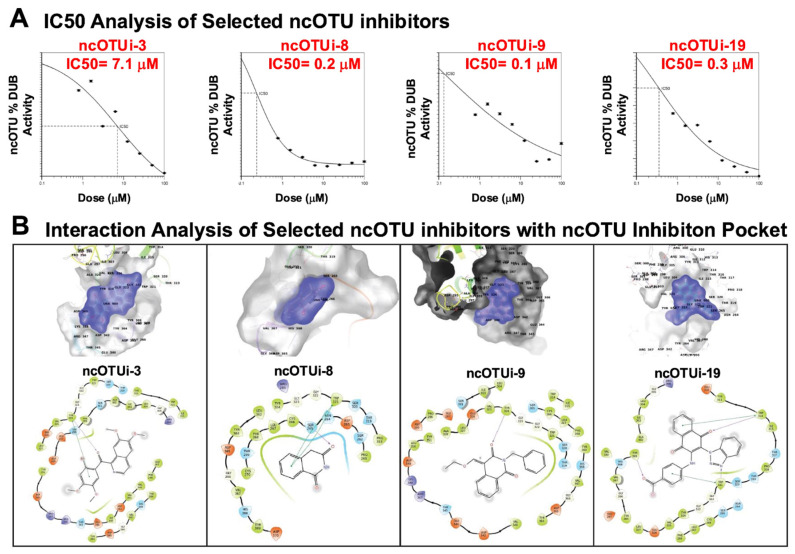
Potency and Binding of ncOTU inhibitors. (**A**) IC50 analysis of selected ncOTU inhibitors. IC50 values were calculated from the fitted curves. Bar graph showing the IC50 values (μM) for compounds ncOTUi-3 (7.1 μM), ncOTUi-8 (0.2 μM), ncOTUi-9 (0.1 μM), and ncOTUi-19 (0.3 μM). (**B**) Interaction analysis of selected ncOTU inhibitors with the ncOTU inhibition pocket. Molecular docking snapshots showing the binding modes of compounds (ncOTUi-3, -8, -9, -19) within the conserved Y-W inhibition pocket of ncOTU.

Dose-response analysis revealed that compounds ncOTUi-9 and ncOTUi-19 were the most potent, with IC50 values of 0.1 μM and 0.3 μM, respectively (Figure 7A). Compound ncOTUi-8 exhibited an IC50 of 0.2 μM, while compound ncOTUi-3 was less potent (IC50: 7.1 μM). These results demonstrate that ncOTU can be effectively inhibited by small molecules at nanomolar to low micromolar concentrations.

Consistent with these data, interaction mapping (Figure 7B) indicates that all four inhibitors occupy the same inhibition pocket, with ncOTUi-9 and ncOTUi-19 exhibiting denser contact networks. ncOTUi-9 engages residues around W315-G316 through combined hydrogen bonding and hydrophobic contacts, whereas ncOTUi-19 shows extended interactions, including aromatic stacking and additional polar contacts with adjacent residues, supporting its high potency despite a slightly weaker IC_50_ than ncOTUi-9. In contrast, ncOTUi-8 maintains a compact but stable interaction pattern, while ncOTUi-3 displays fewer and less optimal contacts, consistent with its reduced activity. These results align structural engagement with functional potency and define a clear framework for rational optimization of ncOTU inhibitors.

## 3. Discussion

Intracellular parasites demonstrate sophisticated strategies to evade host immune defenses, and the subversion of ubiquitin signaling is a common theme among diverse pathogens [18,19]. In this study, we provide the first comprehensive characterization of ncOTU, an OTU-like deubiquitinating enzyme from the apicomplexan parasite *Neospora caninum*. Our results demonstrate that ncOTU is a functional DUB that modulates host cellular ubiquitination and potently suppresses innate immune signaling, identifying it as a critical virulence factor and a promising therapeutic target for neosporosis.

The discovery that ncOTU shares complete identity of the catalytic and inhibition pocket residues, despite only moderate overall sequence similarity with the well-characterized viral OTU protease. This degree of conservation, despite the genetic and biological difference between a virus and an apicomplexan parasite, suggests strong need to have this enzymatic function. Our 3D homology model, validated by Ramachandran analysis and ubiquitin docking studies, confirmed that these residues are positioned appropriately to form a functional active site.

The *in vitro* DUB activity of recombinant ncOTU against UB-AMC substrates provides direct biochemical evidence that ncOTU is indeed a functional deubiquitinase. The enzymatic velocity is comparable to that reported for viral or malarial OTU under similar assay conditions [9,11]. More importantly, the ubiquitome analysis demonstrated that ncOTU expression in mammalian cells significantly reduces both poly- and mono-ubiquitinated protein levels. The preferential effect on mono-ubiquitinated proteins is particularly interesting, as mono-ubiquitination regulates diverse cellular processes including endocytosis, histone regulation, and DNA repair, distinct from the proteasomal degradation role of polyubiquitination [14,20]. This suggests that ncOTU may target specific signaling pathways regulated by mono-ubiquitination.

Our gene expression analysis revealed that ncOTU expression leads to profound suppression of key innate immune genes, particularly *NFKB1* and *IFNA1*. This pattern mirrors the immunosuppressive strategy employed by viruses, where the viral OTU protease blocks NF-κB activation by removing ubiquitin chains from critical signaling intermediates [21].

The observation that *CASP1* was suppressed is particularly revealing. Caspase-1 is a key effector of inflammasomes, responsible for inducing pyroptosis and processing the pro-inflammatory cytokines *IL1β* and *IL18* [22]. This suggests that ncOTU may trigger initial recognition of parasitic DNA (potentially from the parasite’s dense granules or apicoplast but then blocks the downstream pyroptotic cell death pathway by inhibiting caspase-1. Such a strategy would allow the parasite to prevent host cell death while simultaneously suppressing inflammatory cytokine production (IL 1β and IL-8), which are processed by caspase-1.

Host defense against parasitic infections critically depends on proper cytokine-mediated signaling [23] and the activation of Toll-like receptors [24]. The potent suppression of interferon-stimulated genes *APOBEC3G* and *MX1* further supports the conclusion that ncOTU effectively disarms the type I interferon response. *APOBEC3G* is a cytidine deaminase that restricts retroviruses and potentially other pathogens, while *MX1* is a dynamin-like GTPase with broad antiviral activity. Their suppression would create a more permissive environment for intracellular parasite survival.

Neosporosis continues to pose a significant challenge for the livestock industry and veterinary medicine, with recent systematic reviews confirming that treatment options remain severely limited and often ineffective [25]. A nationwide serological study has revealed that *N. caninum* infection is endemic in sheep and cattle, underscoring its substantial economic impact on national livestock production [1,26,27]. The most comprehensive analysis to date, encompassing 82 canine cases, has demonstrated that the duration of antimicrobial therapy is the only consistent prognostic marker for survival, yet relapses are common and complete remissions remain rare [28]. Similarly, in a retrospective study of 21 adult dogs, treatment response to clindamycin was variable with a relapse and a one-year survival rate of only 57% [29]. Although the search for effective chemotherapeutics has intensified, with numerous compounds including piritrexim, monensin, pyrimethamine, and trimethoprim demonstrating efficacy in infected cell cultures, and promising *in vivo* studies of artemisinin and dihydroartemisinin in murine models [24,30,31], a critical translational gap persists. Encouraging progress is also being made in the development of targeted inhibitors; for instance, emodin has recently been shown to directly target *N. caninum*, inhibiting its invasion and proliferation across multiple host cell lines [32]. Moreover, the successful development of first-in-class small molecule inhibitors targeting OTU-like deubiquitinases in *Plasmodium* species also provides a strong rationale for targeting ncOTU as a novel anti-neosporosis strategy [11].

Our discovery of first-in-class small molecule inhibitors targeting ncOTU represents a significant advance. Compounds ncOTUi-8, ncOTUi-9 and ncOTUi-19 inhibited ncOTU with IC50 values of 0.1 μM–0.3 μM potencies in the nanomolar range that are suitable for further development. The observation that these compounds were identified through structure-based virtual screening targeting the conserved Y-W inhibition pocket validates the pocket as a druggable site. Notably, compound ncOTUi-19 exhibited more extensive interactions with ncOTU, including binding to Y369 (adjacent to the catalytic H362) and W314, which may explain its potent activity. These structural insights will guide medicinal chemistry efforts to optimize these lead compounds for improved potency, selectivity, and pharmacokinetic properties. However, the experimental inhibition data show only partial agreement with docking predictions, highlighting the necessity of empirical validation in structure-based virtual screening workflows. This limited correlation may reflect the intrinsic flexibility of the ncOTU active site and the simplified assumptions inherent to docking scoring functions. Overall, inhibitory potency appears more consistently associated with structural features such as increased molecular complexity, aromatic ring density, and the presence of heterocyclic and electron-withdrawing substituents, which may enhance binding interactions despite variability in docking scores. Future work needs to focus on improving predictive performance through approaches such as ensemble docking, molecular dynamics-based refinement, and larger, more diverse compound libraries to enable more robust structure–activity analyses and stronger validation of computational models.

It will be important to assess selectivity against host DUBs. Sequence analysis demonstrated that ncOTU shares only moderate overall similarity with mammalian OTU-family deubiquitinases, including bovine, canine, and human homologs (Figure S3), with sequence identities remaining below 31%. Importantly, while the catalytic core residues are conserved, surrounding residues around the Y-W-G inhibition pocket targeted by the identified compounds display notable divergence from host OTU enzymes. These differences suggest that the inhibitor-binding environment in ncOTU may possess parasite-specific structural features that could potentially be exploited for selective inhibitor design. Although these observations provide a preliminary rationale for reduced host cross-reactivity, comprehensive biochemical selectivity profiling against bovine, canine, and human OTU-family DUBs will still be essential to experimentally validate specificity and minimize potential off-target effects [33].

Several limitations of this study should be discussed. First, our experiments were performed using an overexpression system in HEK293 cells rather than in *N. caninum*-infected cells. While this approach allowed us to characterize ncOTU’s enzymatic and immune-modulatory functions in isolation, future studies should confirm these findings in the context of actual parasite infection using *N. caninum*-infected cell lines or animal models. Second, the cellular targets of ncOTU remain to be identified. While we have demonstrated that ncOTU globally affects the ubiquitome and immune gene expression, the specific host proteins that are deubiquitinated by ncOTU are unknown. Mass spectrometry-based ubiquitin remnant profiling in ncOTU-expressing cells could identify endogenous substrates. Third, the anti-parasitic efficacy of compounds needs to be validated in *N. caninum* culture systems and in animal models of neosporosis. Fourth, our transfection experiments were performed in HEK293 human cells rather than bovine or canine cell lines. While human cells serve as a useful model for studying conserved ubiquitin and innate immune signaling pathways, future studies should confirm these findings in physiologically relevant host cells, including Madin-Darby Bovine Kidney (MDBK) cells or Madin-Darby Canine Kidney (MDCK) cells, to validate that ncOTU similarly modulates immune responses in natural host species. Additionally, comparative sequence analysis revealed substantial divergence between ncOTU and mammalian OTU-family DUBs, particularly within the inhibitor-binding pocket, supporting the rationale for future selective inhibitor development. Fifth, we did not include a catalytically inactive ncOTU mutant (e.g., C260A or H362A) as a negative control in our cellular assays. While our *in vitro* UB-AMC data confirm enzymatic activity, and the immune suppression phenotype is consistent with known DUB-mediated immune evasion, future studies should include catalytic-dead mutants to definitively establish causality between ncOTU’s DUB activity and the observed transcriptional changes. Sixth, our transcriptomic findings were not validated at the protein level (e.g., NF-κB phosphorylation, cytokine secretion). While mRNA changes often correlate with protein levels for these immune related genes, future studies should confirm these results using immunoblotting for phosphorylated proteins, as well as ELISA-based quantification of IFN-β and IL-1β secretion. Moreover, our *in vitro* DUB inhibition data are promising, cellular activity against the parasite and favorable pharmacokinetic properties (oral bioavailability, metabolic stability, and blood-brain barrier penetration for neurological neosporosis) remain to be demonstrated.

Future studies should also determine the kinetic parameters (Km, Vmax, kcat) of ncOTU against defined ubiquitin chain types (K48, K63, linear) and mono-ubiquitin to fully define its substrate preferences. We caution that our conclusions regarding ‘immune evasion’ are limited to the NF-κB and type I interferon pathways in HEK293 cells. The impact of ncOTU on other immune mechanisms including complement fixation, natural killer cell activity, antigen presentation, and phagocyte recruitment remains unknown and requires future investigation in relevant infection models. Finally, the potential for drug resistance development should be assessed. Given that ncOTU is likely essential for parasite virulence, resistance mutations in the OTU domain might compromise parasite fitness, potentially slowing the emergence of clinically significant resistance. Besides, the inhibitors were characterized exclusively in cell-free enzyme assays. Their cytotoxicity, cellular permeability, and anti-parasitic activity in *N. caninum*-infected cells remain unknown. Future studies must evaluate compound safety in host cells (e.g., MDBK or MDCK) and efficacy against intracellular parasites before any preclinical conclusions can be drawn.

Another important limitation of the current study is that the direct cellular substrates and interaction partners of ncOTU remain unknown. Identification of these host targets will be essential for defining the precise molecular mechanisms underlying ncOTU-mediated immune modulation. In future studies, complementary bioinformatics approaches including host–parasite protein interactome prediction, ubiquitination site/motif enrichment analysis, pathway network mapping, and structure-based substrate docking could be used to prioritize candidate host proteins potentially targeted by ncOTU. Such *in silico* predictions could then be experimentally validated using proteomic approaches such as co-immunoprecipitation coupled with mass spectrometry, ubiquitin remnant profiling, or proximity-labeling strategies. These efforts would provide a more comprehensive understanding of ncOTU substrate specificity and its role in *N. caninum* host-pathogen interactions. In addition, recent advances in AI-driven protein structure prediction, including AlphaFold [34,35] and RoseTTAFold [35,36], have significantly transformed structure-based drug discovery, particularly for pathogen proteins lacking experimentally resolved structures [35,37]. Future studies could benefit from integrating such AI-based predictions alongside traditional homology modeling approaches to further validate the ncOTU catalytic and inhibitor binding pocket architecture, thereby improving confidence in structure-based virtual screening results.

In summary, this study provides the first functional characterization of ncOTU, a OTU deubiquitinating enzyme from *Neospora caninum*. We have demonstrated that ncOTU possesses bona fide DUB activity *in vitro* and in mammalian cells, modulates the host cellular ubiquitome, suppresses NF-κB and type I interferon signaling pathways, and can be effectively inhibited by small molecules targeting its conserved Y-W inhibition pocket. The identification of nanomolar inhibitors (ncOTUi-8, ncOTUi-9 and ncOTUi-19) represents a significant step toward the development of targeted therapies for neosporosis. These findings establish ncOTU as a critical virulence factor and a promising molecular target for anti-neosporosis drug discovery.

## 4. Materials and Methods

### 4.1. Codon Optimization and Gene Synthesis

The amino acid sequence of *N. caninum* OTU-like protein (ncOTU; NCBI Reference Sequence: XP_003886403) was retrieved from the NCBI protein database. Codon optimization for expression in *E. coli* was performed using the OPTIMIZER server (http://genomes.urv.es/OPTIMIZER/ accessed on 1 July 2019) with *E. coli* K12 strain parameters, as previously described [9]. The “highly expressed genes” (HEG) codon usage table was selected, and the “guided random” method was employed. A C-terminal 6xHisTag sequence (CATCATCACCATCATCAC) followed by a stop codon (TAA) was added to the optimized nucleotide sequence using ApE plasmid editor software (v3.1.9). Homology arms (15–20 nt) compatible with In-Fusion cloning into pET-26b(+) vector were added, ensuring the absence of internal NdeI, NcoI, XbaI, and EcoRI restriction sites. The final synthetic gene fragments (gBlocks^®^ Gene Fragments, IDT, Newark, NJ, USA) were synthesized and cloned into the pTwist31_Amp vector.

### 4.2. Bacterial Expression Vector Construction

The pTwist31_Amp vectors containing codon-optimized ncOTU sequences were digested with NdeI and NcoI restriction enzymes (New England Biolabs, Ipswich, MA, USA) to release the ncOTU insert. Following agarose gel electrophoresis, the 1437 bp ncOTU fragment was excised and purified using a gel extraction kit (Qiagen, Hilden, Germany). The pET-26b(+) bacterial expression vector (Novagen, Madison, WI, USA, Cat#69862-3) was linearized with the same restriction enzymes (NdeI and NcoI). The purified ncOTU insert was then cloned into the linearized pET-26b(+) vector using the In-Fusion^®^ HD Cloning System (Clontech, San Jose, CA, USA, Cat#639645) according to the manufacturer’s instructions. The resulting recombinant plasmids (pET-26b-ncOTU) were transformed into Stellar competent *E. coli* cells. Positive clones were selected by colony PCR and confirmed by restriction digestion with NdeI and EcoRI, followed by DNA sequencing (Medsantek, Istanbul, Türkiye).

### 4.3. Mammalian Expression Vector Construction

For mammalian expression studies, the ncOTU coding sequence was subcloned from pET-26b-ncOTU into the pcDNA3.1(+) mammalian expression vector (Invitrogen, Carlsbad, CA, USA). Briefly, pET-26b-ncOTU was digested with XbaI (compatible with NheI) and EcoRI. The released ncOTU fragment was gel-purified and ligated into pcDNA3.1(+) that had been linearized with NheI and EcoRI and dephosphorylated using alkaline phosphatase. The resulting pcDNA3.1-ncOTU constructs were verified by restriction digestion and DNA sequencing.

### 4.4. Recombinant Protein Expression

For protein expression, pET-26b-ncOTU was transformed into *E. coli* BL21(DE3) competent cells. Transformants were grown in LB medium containing 50 μg/mL kanamycin at 37 °C until the optical density at 600 nm (OD600) reached 0.6. Protein expression was induced by the addition of 1 mM isopropyl β-D-1-thiogalactopyranoside (IPTG), and cultures were incubated overnight at 25 °C with shaking (200 rpm). Uninduced controls were processed in parallel. Cells were harvested by centrifugation at 4000× *g* for 15 min at 4 °C and stored at −80 °C until further processing.

### 4.5. Protein Purification

Cell pellets were resuspended in solubilization buffer (8 M urea, 20 mM Tris-HCl, pH 8.0, 500 mM NaCl, 20 mM imidazole) and lysed by sonication (5 cycles of 30 s on ice). The lysate was centrifuged at 15,000× *g* for 30 min at 4 °C to remove cellular debris, and the supernatant was filtered through a 0.45 μm syringe filter. Purification was performed using an ÄKTAprime protein purification system (GE Healthcare, Chicago, IL, USA) equipped with a 1 mL HisTrap HP affinity column (GE Healthcare, Cat#17-5248-01). The column was equilibrated with a binding buffer (20 mM Tris-HCl, pH 8.0, 500 mM NaCl, 20 mM imidazole). Following sample loading, the column was washed with a wash buffer (20 mM Tris-HCl, pH 8.0, 500 mM NaCl, 40 mM imidazole) until the UV absorbance returned to baseline. Bound proteins were eluted with an elution buffer (20 mM Tris-HCl, pH 8.0, 500 mM NaCl, 500 mM imidazole). Eluted fractions containing ncOTU were pooled and desalted using Amicon Ultra-0.5 mL centrifugal filters (10,000 NMWL, Millipore, Bedford, MA, USA) and buffer-exchanged into PBS (pH 7.4).

### 4.6. Protein Refolding and Solubility Analysis

Desalted ncOTU was assessed for proper folding using the QuickFold™ Protein Refolding Kit (Athenaes, Baltimore, MD, USA, Cat#0600). Briefly, 25 μL of purified ncOTU was added to 475 μL of 15 different refolding buffers and incubated for 1 h at 4 °C. Samples were centrifuged at 14,000× *g* for 5 min to separate soluble (properly folded) from insoluble (misfolded/aggregated) protein. Supernatant and pellet fractions were analyzed by SDS-PAGE. Protein concentrations in both fractions were measured using a NanoDrop™ 2000 spectrophotometer (Thermo Scientific, Wilmington, DE, USA) at A280 nm. Solubility factor was calculated as: [supernatant protein]/[supernatant + pellet protein] × 100.

### 4.7. Protein Concentration Determination

Protein concentrations were determined using the bicinchoninic acid (BCA) assay (Pierce™ BCA Protein Assay Kit, Thermo Scientific, Waltham, MA, USA). Bovine serum albumin (BSA) standards (0, 0.025, 0.125, 0.25, 0.5, 0.75, 1, 1.5, and 2 mg/mL) and ncOTU samples were prepared in PBS. Following the manufacturer’s protocol, absorbance was measured at 562 nm using a Varioskan™ LUX multimode microplate reader (Thermo Scientific, Waltham, MA, USA). Concentrations were calculated from the standard curve.

### 4.8. SDS-PAGE and Western Blot Analysis

Protein samples were mixed with a 2× Laemmli sample buffer, boiled for 5 min, and resolved on 12% SDS-polyacrylamide gels. Gels were either stained with Coomassie Brilliant Blue R-250 or transferred to PVDF membranes (Millipore, Bedford, MA, USA) for Western blot analysis. For anti-HisTag detection, membranes were blocked with 5% non-fat milk in TBST (Tris-buffered saline with 0.1% Tween-20) for 1 h at room temperature, then incubated with mouse anti-HisTag primary antibody (Pierce, Waltham, MA, USA, 1:1000) overnight at 4 °C. After washing, membranes were incubated with HRP-conjugated secondary antibody (1:2000) for 1 h at room temperature. Signal was detected using Pierce ECL Plus reagent (Thermo Fisher Scientific, Rockford, IL, USA) and visualized with a Bio-Rad ChemiDoc MP imaging system (Bio-Rad Laboratories, Hercules, CA, USA). For ubiquitin detection, transfected HEK293 cells were lysed in RIPA buffer (Santa Cruz, Dallas, TX, USA) containing PMSF. Following SDS-PAGE and transfer, membranes were probed with anti-polyubiquitin (BML-PW8805, Enzo Life Sciences, Farmingdale, New York, NY, USA) or anti-mono/polyubiquitin (BML-PW8810, Enzo Life Sciences, Farmingdale, New York, NY, USA) antibodies (1:1000). Beta-actin was used as a loading control.

### 4.9. Cell Culture and Transfection

HEK293 human embryonic kidney cells were cultured in DMEM supplemented with 10% fetal bovine serum (FBS), 100 U/mL penicillin, and 100 μg/mL streptomycin at 37 °C in a 5% CO_2_ humidified incubator. For transfection, 4 × 10^5^ cells were seeded into 6-well plates 24 h prior to transfection. Cells were transfected with 2 μg of pcDNA3.1-ncOTU or empty pcDNA3.1 vector (negative control) using 2 μg of polyethylenimine (PEI) in 200 μL of serum-free DMEM. Transfection efficiency was monitored using a GFP-expressing plasmid. Cells were harvested 72 h post-transfection for downstream analyses.

### 4.10. RNA Isolation and Real-Time PCR

Total RNA was isolated from transfected HEK293 cells using NucleoZOL reagent (Macherey-Nagel, Düren, Germany, REF 740406.50) according to the manufacturer’s protocol. First-strand cDNA was synthesized from 1 μg of total RNA using the ProtoScript II First Strand cDNA Synthesis Kit (New England Biolabs, Ipswich, MA, USA, Cat# E6560). Real-time PCR was performed using a LightCycler 96 system (Roche, Mannheim, Germany) with SYBR Green Master Mix (Applied Biosystems, Foster City, CA, USA). Primers for target genes were obtained from the PrimerBank (https://pga.mgh.harvard.edu/primerbank/ accessed on 1 July 2019) (Table 3). GAPDH was used as an internal control for normalization. The thermal cycling conditions were: 95 °C for 2 min, followed by 55 cycles of 95 °C for 15 s, 60 °C for 60 s, and 72 °C for 45 s. Relative gene expression was calculated using the ΔΔCt method and normalized to the empty vector control (DMSO control group).

### 4.11. Homology Modeling, Molecular Docking and Compound Selection

The three-dimensional structure of ncOTU was generated by homology modeling using the SWISS-MODEL web server (https://swissmodel.expasy.org). The amino acid sequence of ncOTU (XP_003886403) was used as the query, and the crystal structure of CCHFV OTU protease (PDB ID: 3PRP.A) was selected as the template based on sequence similarity. The resulting model was visualized and analyzed using Maestro (Schrödinger Suite, 2020-1, New York, NY, USA). Structural validation was performed using Ramachandran plots and RMSD (root mean square deviation) calculations. Protein-protein docking between ncOTU and human ubiquitin (PDB ID: 3PHW.B) was performed using the PIPER docking protocol within the Schrödinger Biologics Suite (2020-1, New York, NY, USA). The resulting complexes were visualized and analyzed for interactions with conserved catalytic and inhibition pocket residues using AutoDockTools-1.5.6. A small molecule library was assembled from PubChem and ZINC databases based on drug-like properties with known OTU inhibition as we reported previously [9,10,11]. Selected small molecules were screened against the ncOTU homology model using AutoDock Vina (autodock_vina_1_1_2_linux). A docking grid box (22 × 20 × 20 Å) was centered on the predicted inhibition pocket (Y-W-G). Compounds were purchased from Molport (New York, NY, USA).

### 4.12. In Vitro Deubiquitinase Activity and Inhibition Assays

Deubiquitinase activity of recombinant ncOTU was measured fluorometrically using Ubiquitin-AMC (UB-AMC) substrate (Boston Biochem, Cambridge, MA, USA). Standard reactions were performed in 10 mM HEPES (pH 7.5), 100 mM NaCl, and 2.5 mM DTT. Reactions contained 1 μM UB-AMC and 1 μM purified ncOTU protein in a final volume of 100 μL. Fluorescence was measured at 5-min intervals for 60 min at 25 °C using a Varioskan™ LUX microplate reader (Thermo Scientific, Waltham, MA, USA) (excitation 345 nm, emission 445 nm). Initial enzymatic velocities were calculated from the linear portion of the progress curves. For inhibitor screening, compounds were tested at 20 μM final concentration (0.1% DMSO). ncOTU was pre-incubated with inhibitor for 10 min at room temperature before addition of UB-AMC substrate. Reactions were initiated by substrate addition, and fluorescence was monitored as described above. Ribavirin (a non-inhibitor control) and DMSO (vehicle control) were used as controls. Percent inhibition was calculated relative to untreated controls. For IC50 determination, dose-response curves were generated using inhibitor concentrations ranging from 0.01 to 100 μM. IC50 values were calculated using AAT Bioquest IC50 Calculator (linear regression with log transformation).

### 4.13. Statistical Analysis

All experiments were performed at least in triplicate (*n* = 3) otherwise indicated. Data are presented as mean ± standard error (SE). Statistical significance between groups was determined using Student’s *t*-test. A *p*-value < 0.05 was considered statistically significant (* *p* < 0.05, ** *p* < 0.01).

## Figures and Tables

**Table 1:** Conserved catalytic and inhibition residues in ncOTU relative to CCHFV OTU (sequence identity at these specific positions).

Protein	Catalytic Residues	Inhibition Pocket Residues
CCHFV OTU	D37, C40, H151	Y89, W99, G100
ncOTU	D257, C260, H362	Y305, W315, G316

**Table 2:** SMILES and calculated binding energies of studied compounds against ncOTU.

ncOTU ID	SMILES	Binding Energy (kcal/mol) ncOTU
ncOTUi-1	C1=CC=C2C(=C1)C(=O)C(=C(C2=O) N3C4=CC=CC=C4N=N3)NC5=CC=CC(=C5)C(=O)O	−7.5
ncOTUi-2	C1C2=CC=CC=C2C(=O)N(C1=O) CCC3=CC=CC=C3	−6.2
ncOTUi-3	COC1=C(C=C2C(=C1)C=CN=C2C(=O) C3=CC(=C(C=C3Br)OC)OC)OC	−5.8
ncOTUi-4	C1=CC=C(C(=C1) CNC2=CC=CC3=C2N=CC=C3)O	−6.4
ncOTUi-5	C1CCCN(CC1)C(=S) NC2=CC3=NSN=C3C=C2	−5.9
ncOTUi-6	CCOC1=CC=C(C=C1)NC(=S) NC2=CC3=NSN=C3C=C2	−5.8
ncOTUi-7	COC1=C2C3=C (C4=CC=CC=C4C2=O)ON=C3C=C1	−6.7
ncOTUi-8	C1C2=CC=CC=C2C(=O)NC1=O	−5.1
ncOTUi-9	CCOC=C1C2=CC=CC=C2C(=O)N (C1=O)CC3=CC=CC=C3	−6.3
ncOTUi-10	C1C2=CC=CC=C2C(=O)N(C1=O) CC3=CC=CC=C3	−6.3
ncOTUi-11	CCCCN1C(=C (C2=NC3=CC=CC=C3N=C21)C#N)NC (=O)C4=CC=CC=C4	−6.4
ncOTUi-12	CC1=CC=C(C=C1)N2C(=O)C3C(C2=O) C(N4C3C5=CC=CC=C5C=C4)C(=O) C6=CC=CO6	−7.9
ncOTUi-13	C1=CC=C2C(=C1)C(OC2=O)NC3=C4C (=CC(=C3)Br)C=CC=N4	−6.9
ncOTUi-14	CC(=O)C1=CC=C(C=C1)NC(=S) NC2=CC=CC3=C2C=CN=C3	−6.2
ncOTUi-15	C1=CC2=C(C=CN=C2)C(=C1)NC(=S) NC3=CC=C(C=C3)Cl	−6.1
ncOTUi-16	C1=CC=C2C3C4C(C(N3C=CC2=C1)C (=O)C5=CC=CO5)C(=O)N(C4=O) C6=CC=C(C=C6)Br	−7.7
ncOTUi-17	COC1=CC=C(C=C1)C=NNC(=O) C2=NN(C(=O)C3=C(SC=C32)N) C4=CC=CC=C4	−6.6
ncOTUi-18	CC(=O)C1C2C (C3N1C=CC4=CC=CC=C34)C(=O)N (C2=O)C5=CC=C(C=C5)OC	−6.8
ncOTUi-19	C1=CC=C2C(=C1)C(=O)C(=C(C2=O) N3C4=CC=CC=C4N=N3)NC5=CC=C (C=C5)C(=O)O	−6.7
ncOTUi-20	CN1C2=CN(C(=C2C(=O)N(C1=O)C) C3=CC=CC=C3)C4=CC=C(C=C4)N	−6.4

**Table 3:** List of primers.

Gene	Forward Primer (5′–3′)	Reverse Primer (5′–3′)
*AIM2*	GCAGTGATGAAGACCATTGC	GGCTTTGGTTTTGTTACCGGA
*CASP1*	ATCGCTTCTTCTGCTTCCAC	AATGAAAATCGAACCTTGCG
*IL18*	TGCAGTCTACACAGCTTCGG	ACTGGTTCAGCAGCCATCTT
*IL1B*	GAAGCTGATGGCCTCAAACA	AAGCCCTTGTGTAGGTGGT
*cGAS*	TGGCTTTCAGCAAAAGTTAGG	AAGGATAGCCGCCATGTTTC
*STING*	GATATCTGCGGCTGATCCTG	ATATACAGCCGCTGGCTCAC
*TBK1*	GCAGTTTGTTTCTCTGTATGGC	AATGTTACCCCAATGCTCCA
*IRF3*	ATGCACAGCAGGAGGATTTC	GTTGGCAGGTCTGGCTTATC
*TLR3*	AGGAAAGGCTAGCAGTCATCC	GCTGCAGTCAGCAACTTCAT
*RIG-I*	ATATCCGGAAGACCCTGGAC	GAGAAAAAAGTTGGGCAGCCT
*MDA5*	TTCAACCACAGTTCAGCCAA	TGACACTTCCTTCTGCCAAA
*MAVS*	GGTCGCCAGGTCTCAGG	TGTCTTCAGCAAACGGCAT
*NFKB1*	ATGTATGTGAAGGCCCATCC	ATAACCTTTGCTGGTCCCAC
*IFNA1*	CAGAGTCACCCATCTCAGCA	CTTGACTTGCAGCTGAGCAC
*IFNA2*	GCTCACCCATTTCAACCAGT	CTTGACTTGCAGCTGAGCAC
*IFNAR1*	GACCCTAGTGCTCGTGC	ACTCATCGCTCCTGTTCACC
*IFNB1*	CTTTGGAAGCCTTTGCTCTG	CAGGAGAGCAATTTGGAGGA
*STAT1*	TTCAGGAAGACCCAATCCAG	TGCTCTGAATATTCCCCGAC
*APOBEC3G*	AGGGGCTTTCTATGCAACC	TTCCAAAAGGGAATCACGTC
*G1P2*	GCGAACTCATCTTTGCCAGT	AGGGACACCTGGGAATTCGTT
*IL15*	AGAAGCCAACTGGGTGAATG	ACTTTGCAACTGGGGTGAAC
*MX1*	GATTTTGGGGCTTTCCAGTC	GATGATCAAAGGGATGTGGC

## Data Availability

The original contributions presented in this study are included in the article and Supplementary Material. Further inquiries can be directed to the corresponding author.

## Supplementary Materials

The following supporting information can be downloaded at: https://www.mdpi.com/article/10.3390/ijms27125178/s1.
