# Supplementary material for: Functional Characterization of a Novel OTU-like Deubiquitinase from Neospora caninum and Discovery of Small-Molecule Inhibitors
Source: Int J Mol Sci. 2026 Jun 7;27(12):5178. doi: 10.3390/ijms27125178 (PMC13299058; doi:10.3390/ijms27125178)
Supplement: Supplementary file 1 [file ijms-27-05178-s001.zip › ijms-4286869-supplementary.pdf]

Supplementary Figures

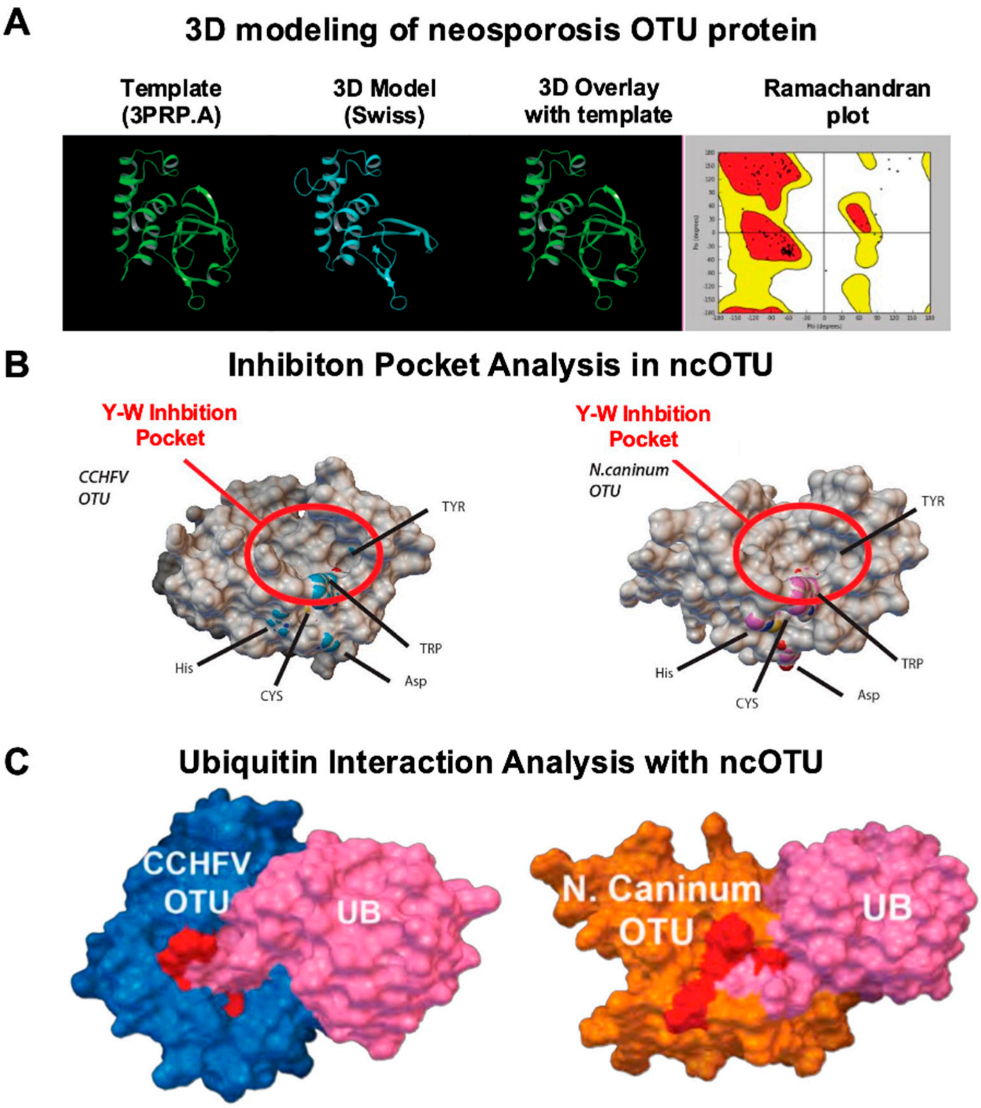

Figure S1. Homology modeling, inhibition pocket and ubiquitin interaction analysis of ncOTU. (A) 3D homology model of ncOTU generated using SWISS-MODEL with CCHFV OTU (PDB: 3PRP.A) as template. (B) The CCHFV OTU and ncOTU model is shown with the inhibition pocket highlighted. Close-up view of the ncOTU active site showing the conserved catalytic triad (His, Cys, Asp) and inhibition pocket residues (Tyr, Trp) as sticks. C) Ubiquitin Interaction Analysis with CCHFV and ncOTU showing that Ubiquitin interacts through Y-W inhibition pocket.

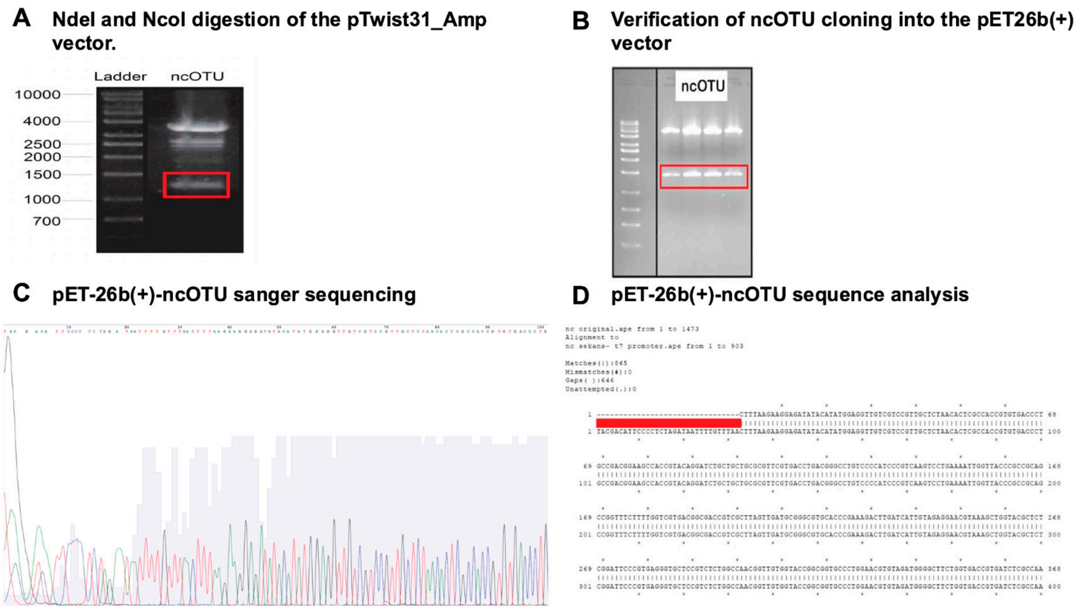

Figure S2. Restriction and sequence analysis for the cloning of ncOTU. (A) Agarose gel electrophoresis of pTwist31_Amp vector digested with NdeI and NcoI restriction enzymes. (B) Agarose gel electrophoresis of pET-26b-ncOTU recombinant clones. The ncOTU insert is indicated by the red box. Snapshot of (C) pET-26b(+)-ncOTU sanger sequencing and (D) pET-26b(+)-ncOTU sequence analysis.

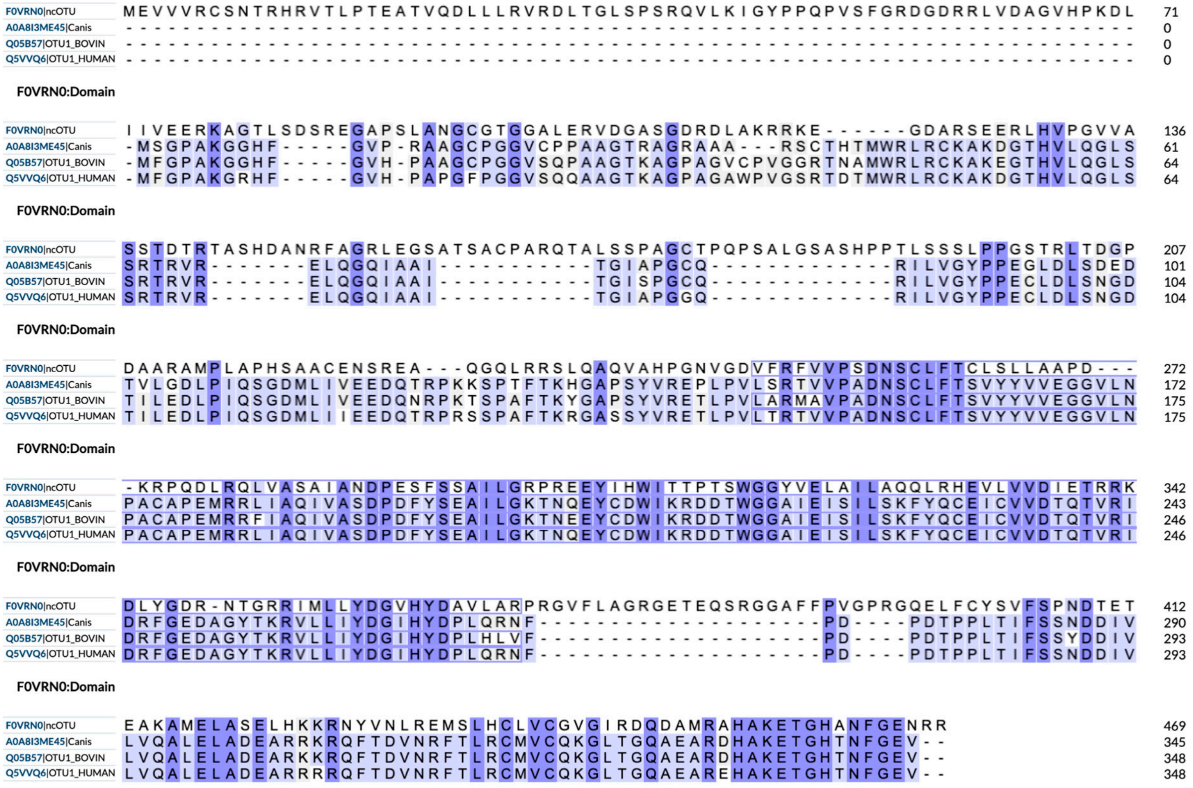

Figure S3. Sequence identity analysis of ncOTU with mammalian OTU-family deubiquitinases. Multiple sequence alignment of *Neospora caninum* ncOTU (XP_003886403; UniProt ID: F0VRN0) with OTU-family homologs from *Canis familiaris* (Canis OTU; XP_038526772.1, UniProt ID: A0A8I3ME45), *Bos taurus* (OTU1_BOVIN; NP_001073778.1,

UniProt ID: Q05B57), and *Homo sapiens* (OTU1_HUMAN; NP_001263249.1, UniProt ID: Q5VVQ6) generated using the UniProt Align tool. Conserved residues are highlighted in blue. Pairwise sequence comparison revealed moderate overall conservation between ncOTU and mammalian OTU-family proteins, with 37.8% similarity and 30.8% identity to canine OTU, 37.4% similarity and 29.9% identity to bovine OTU1, and 34.8% similarity and 28.7% identity to human OTU1. These findings indicate that while catalytic OTU-domain features are conserved, ncOTU remains substantially divergent from mammalian homologs at the overall sequence level.
